# Subtidal Benthic Invertebrates Shifting Northward Along the US Atlantic Coast

**DOI:** 10.1007/s12237-017-0236-z

**Published:** 2017-11

**Authors:** Stephen S. Hale, Henry W. Buffum, John A. Kiddon, Melissa M. Hughes

**Affiliations:** 1 Atlantic Ecology Division, National Health and Environmental, Effects Research Laboratory, Office of Research and Development, US Environmental Protection Agency, Narragansett, RI, USA; 2 CSRA, US Environmental Protection Agency, Narragansett, RI, USA

**Keywords:** Benthic invertebrates, Species’ range shifts, Climate change, US Atlantic coast, Carolinian Biogeographic Province, Virginian Biogeographic Province

## Abstract

Numerous marine and terrestrial species have shifted their ranges poleward in response to warming from global climate change. However, few studies have examined range shifts of subtidal benthic communities in estuarine and nearshore waters. This study examined 20 years (1990–2010) of occurrence and abundance data of soft-bottom, benthic invertebrates along the Atlantic coast of the USA. Data from two biogeographic provinces (Carolinian and Virginian), which spanned 15° of latitude from mid-Florida to Cape Cod, were extracted from a national coastal assessment program. Mean water temperatures increased significantly during the study period, bottom water by 1.6 °C and surface water by 1.7 °C. Of 25 species with significant changes in centers of abundance (out of the 30 most prevalent), 18 (60%) shifted northward and 7 (23%) shifted southward. Species that shifted north moved an average distance of 181 km, in contrast with 65 km for species that shifted south. The southern limits of 22 species showed significant northward shifts; because there was little change in northern limits, this resulted in an average 25% range contraction. Community composition changed during the study period, most notably in southern latitudes. Five Carolinian species surmounted their northerly biogeographic boundary. Consequences of these range shifts include changes in benthic community structure and function, which have strong implications for ecosystem functioning and services including changes in fisheries dependent upon benthic prey.

## Introduction

Shifts in species’ spatial distributions are one of the expected outcomes of climate change (IPCC 2014). Global warming has forced numerous marine and terrestrial species around the world to shift their ranges poleward (Wernberg et al. 2012; Pinsky et al. 2013; Poloczanska et al. 2013; Sunday et al. 2015). Field, experimental, and modeling studies have established that ocean warming is a dominant factor in observed range shifts (Hawkins et al. 2008; Wernberg et al. 2012; Pinsky et al. 2013; Hiddink et al. 2015; Sunday et al. 2015; Hare et al. 2016). Changes in species’ ranges can have profound effects on ecosystem functioning and services, including adverse impacts on fisheries (Nye et al. 2009; Hawkins et al. 2008; Johnson et al. 2011; Mills et al. 2013; Pershing et al. 2015; Hare et al. 2016). These range shifts are expected to affect marine systems for the foreseeable future (Hare et al. 2016) and could be one of the most significant consequences of global climate change (Hiddink et al. 2015), with potential widespread socioeconomic implications for communities that depend on estuarine and coastal fisheries.

Species tend to retreat from areas that become too warm and expand into areas that were previously too cold (Sunday et al. 2012). At the leading and trailing edges of species’ ranges, where thermal tolerances are reached, temperature is the predominant factor controlling many of their distributions (Jones et al. 2012). To maintain their thermal niches in warming waters, organisms have to track the rate of isotherm movement (Burrows et al. 2011). For the benthos, bottom water temperatures affect settling larvae, juveniles, and adults; surface water temperatures affect pelagic eggs and larvae. The effects of temperature on reproductive success and offspring survival may play a greater role than temperature tolerances of adults in determining population response to warming waters (Engle and Summers 1999). Some benthic species move their ranges via pelagic eggs and larvae, while others simply cease to grow, reproduce, and survive in southern areas that exceed their temperature preferences (Hiscock et al. 2004; Rilov 2016). Benthic invertebrates are less able than fishes to keep pace with rapid shifts in water temperature (Hinz et al. 2011; Poloczanska et al. 2013; Hiddink et al. 2015; Sunday et al. 2015; Hare et al. 2016). If these species cannot keep up with the pace of changing temperatures, a decrease in biodiversity will eventually result (Hiddink et al. 2015).

The US Atlantic coast is experiencing relatively rapid climate change (Melillo et al. 2014). Ocean surface temperatures in this area are expected to rise 1–5 °C by 2100 (IPCC 2014). While several studies have examined the impacts of rapidly warming waters on distributional changes of coastal fishes (Nye et al. 2009; Mills et al. 2013; Pershing et al. 2015; Hare et al. 2016), fewer studies have looked at subtidal, soft- bottom, benthic invertebrate communities (Hinz et al. 2011; Wernberg et al. 2012; Hiddink et al. 2015; Weinert et al. 2016). Soft-bottom habitats are one of the most widespread habitats on Earth and one with many keystone species and ecosystem engineers that play critical roles in biogeochemical cycles, energy transfer to important commercial fisheries, and provision of other essential ecosystem services (Snelgrove 1999). Impacts to these species can have cascading ecological effects such as major changes in food webs and energy flow (Johnson et al. 2011; Smale and Wernberg 2013).

**Fig. 1:**
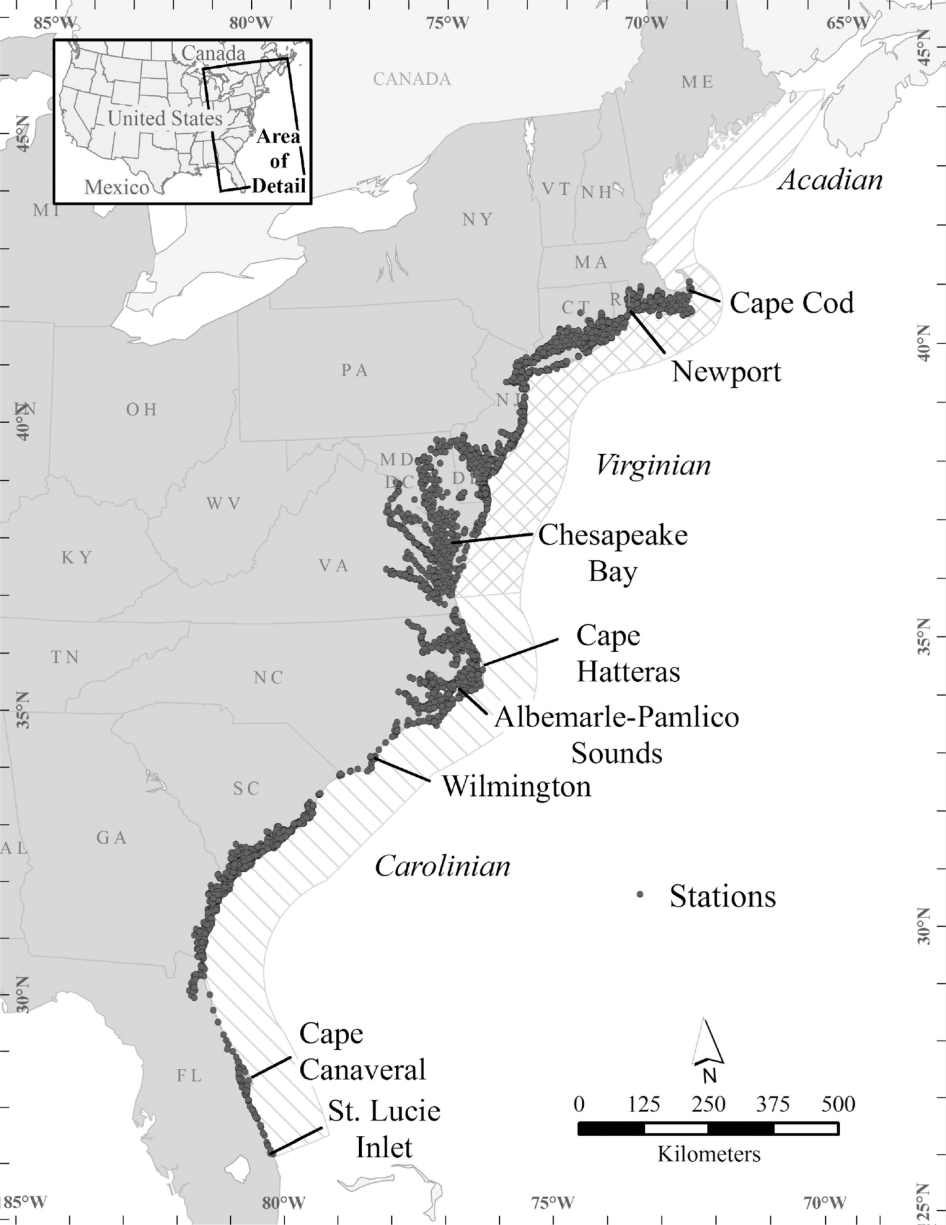
Map of US Atlantic coast showing the Carolinian (CP) and Virginian (VP) and Acadian biogeographic provinces and sampled stations, 1990–2010, in the CP and VP

We examined a 20-year period of occurrence and abundance data for estuarine and nearshore, subtidal, soft-bottom marine benthic invertebrates from two biogeographic provinces spanning 15° of latitude along the Atlantic coast of the USA from mid-Florida to Cape Cod, MA (Fig. 1). We tested the hypotheses that during the period 1990–2010 along the US Atlantic coast, (1) water temperatures rose and (2) benthic invertebrate species’ distributions shifted north.

## Methods

### Study Area and Data

We obtained data from three US Environmental Protection Agency (EPA) coastal assessment programs that used similar sampling design and methods: Environmental Monitoring and Assessment Program (EMAP-Estuaries; NCA 2016), National Coastal Assessment (NCA 2016), and National Coastal Condition Assessment (NCCA 2016). EMAP and NCA were research programs to develop a monitoring program, which became the NCCA (begun in 2010 and scheduled to repeat periodically). Data were collected by EPA, National Oceanic and Atmospheric Administration (NOAA), and state field crews. Standardized protocols were used for sample collection and analysis, and all crews and labs followed the quality control/quality assurance procedures in the National Coastal Condition Assessment’s Quality Assurance Project Plan (USEPA 2010). All three programs used a probabilistic sampling design that each year randomly assigned stations (Fig. 1). Samples were collected during a summer index period (July through September) when it was assumed that certain stresses (e.g., hypoxia, elevated temperature) to the benthic community would be high. The bulk of the sampling was done in late July and early August. Benthic macroinvertebrate assemblages were sampled using a 0.04-m^2^ Young-modified van Veen grab, and samples were sieved with a 0.5-mm mesh screen. Concurrent sediment samples were collected and analyzed for grain size, organic matter content, chemical contaminants, and toxicity. Surface (0.5 m) and bottom (0.5 m above the bottom) water column samples were analyzed for physical-chemical properties (e.g., temperature, salinity, pH, dissolved oxygen, nutrients). Temperature was measured with either a SeaBird CTD, Hydrolab Surveyor, or YSI Sonde. The 1990–2006 data are available at USEPA (2016a) and the 2010 data at USEPA (2016b).

We chose for our study area the Carolinian (CP) and the Virginian (VP) biogeographic provinces, which had the longest time series (Fig. 1). The VP was sampled 1990–1993, the CP 1994–1997, and both provinces 2000–2006 and 2010. Henceforth, we refer to data extracted from the combined EMAP-NCA-NCCA studies as the “combined dataset.” The traditional boundaries of the Carolinian Province are Cape Canaveral, Florida, and Cape Hatteras, NC; the northern boundary of the Virginian Province is Cape Cod, MA (Briggs 1995; Spalding et al. 2007). We used the biogeographic boundaries defined by EMAP, which moved the northern boundary of the CP north so as not to split the estuarine Albemarle-Pamlico Sound system between two provinces (Engle and Summers 1999). Similarly, EMAP moved the southern boundary of the CP south from Cape Canaveral so as not to split the Indian River Lagoon system (Fig. 1).

### Statistical Analyses

We used only taxa identified to species level. Species names were first validated with the Integrated Taxonomic Information System (ITIS 2016) and then, for species not in the ITIS database, with the World Register of Marine Species (WoRMS 2016). All the taxonomy labs doing identifications and counts were required to follow the same quality control/quality assurance procedures (USEPA 2010). Over all the years 1990–2010 and 3194 stations (one grab per station), the study area had 1092 taxa identified to species level. The 30 most prevalent species (found at the most stations) and their abundances are shown in Table 1.

For the analyses of temperature changes and range shifts over time, to eliminate potential latitude bias in the distribution of stations across years, we standardized the data to ensure that an equal number of stations was used for each year within each 1° latitude band. For each 1° latitude band, we used all of the stations from the year that had the fewest stations; then, we randomly selected that same number of stations from all the other years. After standardization of stations by latitude bands, we retained for the analysis 800 stations with 787 taxa identified to species level.

We calculated centroids of latitude and longitude as a measure of shifting spatial distributions. However, because the coast in the study area lies predominantly along a north-south axis, and a regression of mean longitudes of the 30 most prevalent species by year did not show a significant (*p* < 0.05) change over the 20 years, we focus here on changes in latitude. Eastward shifts of estuarine species with a strong preference for oligohaline and mesohaline waters to the deeper and cooler—but more saline—waters of the continental shelf would be limited.

### Changes in Water Temperature

For the mean surface and bottom water temperature data taken concurrently with the combined dataset benthic grabs, we ran a test for normality (Kolmogorov-Smirnov statistic) on the residuals and found that the data did not meet the criteria. Therefore, we used the non-parametric Spearman rank-order correlation (SAS ver. 9.4). Additionally, to show the distribution of temperature changes over the study area, we calculated the mean temperatures in all 1° bands of latitude. Then, we compared the mean surface (and bottom) water temperature of the first and last sampling years with a two-sided *t* test (338 stations).

To put these temperature trends based on data from a probabilistic sampling design into the perspective of a continuous time series at fixed stations in the study area, we plotted the mean summer water temperature for the 3-month (July- September) period from National Oceanic and Atmospheric Administration (NOAA 2016) tide gauge stations at Wilmington, NC (CP) and Newport, RI (VP). We began the series at the point in the mid-1990s when NOAA switched to digital thermometer sensors. Before that time, several instances of missing values potentially biased the results. We then regressed temperature on year, as these data met the Kolmogorov-Smirnov test for normality of residuals.

### Changes in Species’ Mean Latitudes over Time

**Fig. 2:**
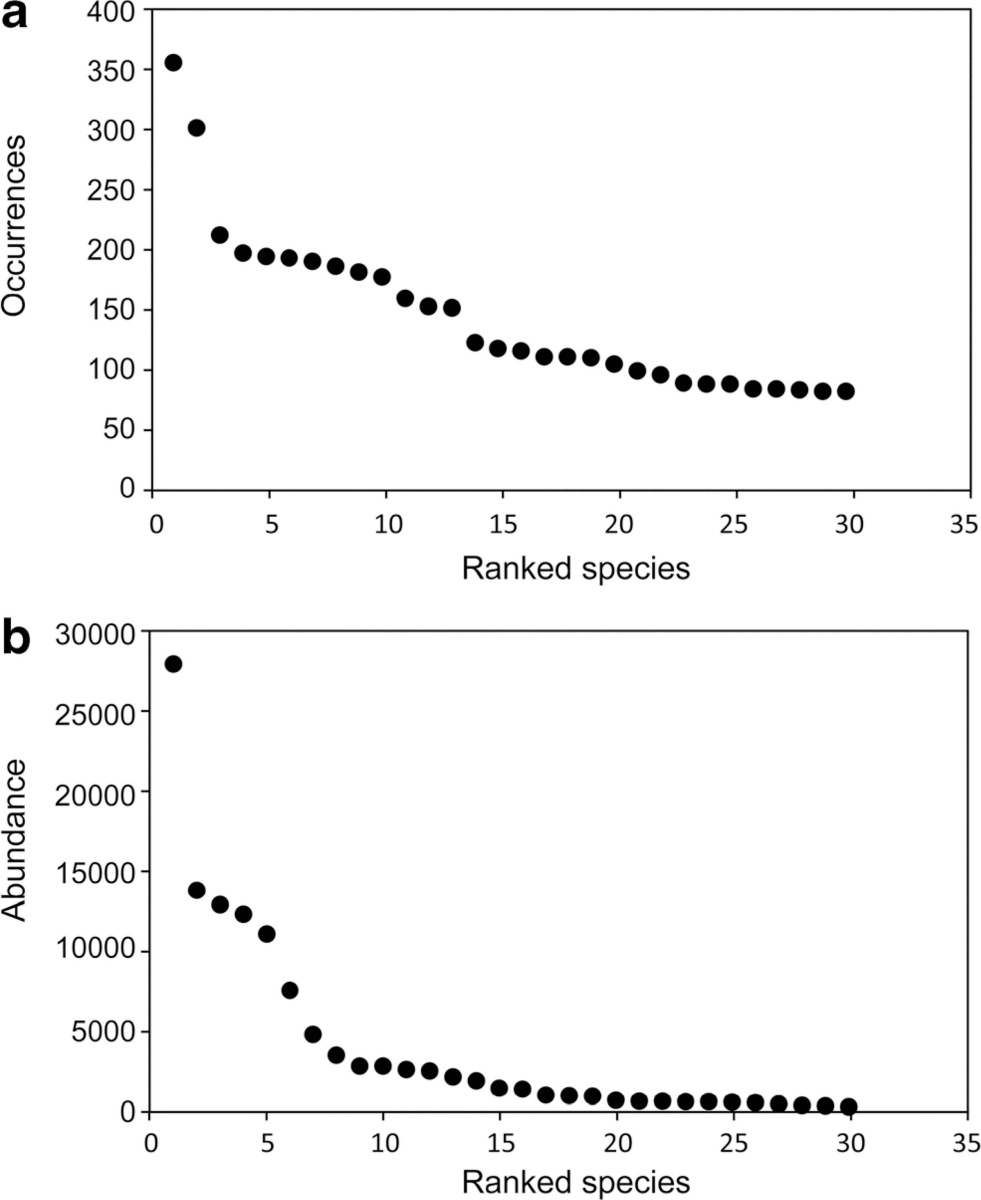
Rank order of the top 30 benthic species in the Carolinian and Virginian biogeographic provinces, 1990–2010. **a** By number of occurrences (3194 stations). **b** By abundance (one 0.04-m^2^ grab per station; 0.5-mm sieve)

To detect changes in mean latitude and minimum and maximum latitude, we limited our analysis to the 30 most prevalent species in the study area (Table 1). We chose 30 based on Fig. 2a, the ranked occurrence of species. The 30th ranked species occurred at about 20% of the stations. At 30, the curve appears to have flattened. Beyond that, there is a greater likelihood that less prevalent species that were actually present in an area would be missed by the sampling program. The rarer the species, the lower our confidence that observed absences were true absences. To check what difference taking a different number would make, we looked at the number of significant shifts in centers of abundance of the 20 most prevalent and the 40 most prevalent species. Each of these gave essentially the same results as 30. Additionally, because we used only the 30 most prevalent species in the analyses of centers of abundance versus time, misidentifications by certified labs would be relatively rare.

To examine changes in the most common 30 species’ distributions over time, we used the eight years (1993/1994, 2000, 2001, 2003–2006, 2010) in which the same spatial extent of both biogeographic provinces had been sampled. We created the earliest year data point by merging the first year of the CP (1994) with the last year of the VP (1993). For each of the 30 most prevalent species, we calculated centers of abundance by weighting the latitude of occurrence by the log10 (X +1) abundance at that station. The relationship between centers of abundance and year did not meet a Kolmogorov- Smirnov test of normality for all 30 species. Therefore, we examined this relationship by calculating non-parametric Spearman rank-order correlations. Distances moved between the first and last year were calculated for the species that had significant Spearman correlations.

### Changes in Species’ Minimum and Maximum Latitudes

To examine potential shifts in the northern and southern limits of species in the study area, we used the occurrence data and determined how many of the 30 most prevalent species in the study area showed a different northern extent (maximum latitude) in 2010 than they did in 1993/1994 and how many showed a different southern extent (minimum latitude). We then applied a sign test (SAS ver. 9.4) to check for statistical significance under the null hypothesis that the median latitudinal movement of the northern and southern limits of the 30 species was 15 (i.e., equal numbers moved north and south). Also, we used quantile regression (SAS ver. 9.4) to calculate the 10th quantile of each species’ centers of abundance by year. We took the 10th quantile to represent the southern extent of the distribution.

### Multidimensional Scaling of Abundances of All Species

As individual species shift their centers of abundance, community composition at the leading and trailing edges would be expected to change. Using all 1092 species from all stations in all years, we ran a non-metric multidimensional scaling (NMDS) in PRIMER 7 (Clarke et al. 2014) on a fourth root- transformed, Bray-Curtis similarity matrix of mean abundances. To check for significant differences among the resulting groups, we ran an analysis of similarity (ANOSIM) comparing provinces and also province-decade pairs. Our expectation was that if large numbers of species shifted their centers of abundance northward, then the community composition of the northerly province in the later years should start to more closely resemble the southerly province in the earlier years.

### Species’ Shifts to a More Northerly Biogeographic Province

Using occurrence data from all 1092 species from all station and all years, we looked for evidence of species extending their range into a different biogeographic province during the study period. We identified CP species that were not found in the VP in 1990 but were found in the VP in 2010. A similar analysis was done for species potentially moving southward from the VP to the CP. To assess the likelihood of false absences for the species that met these criteria, we checked the species ranges given by the World Register of Marine Species (WoRMS 2016), the Ocean Biogeographic Information System (OBIS 2016), the Global Biodiversity Information Facility (GBIF 2016), the Northwest Atlantic Register of Marine Species (NWARMS 2016), and the Chesapeake Bay Program Baywide Benthic Database (Chesapeake Bay Program 2016).

## Results

### Increase in Water Temperatures

**Fig. 3:**
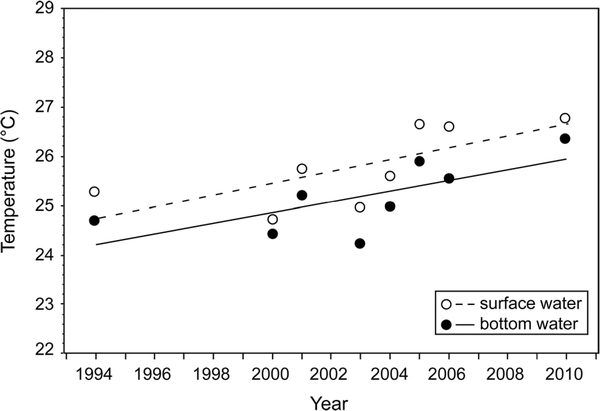
Mean summer (July-September) bottom and surface water temperatures (taken concurrently with benthic samples) and trend lines in the Carolinian and Virginian biogeographic provinces, 1990–2010. Spearman correlations between temperature and year for each depth layer were significant (*p* <0.01)

**Fig. 4:**
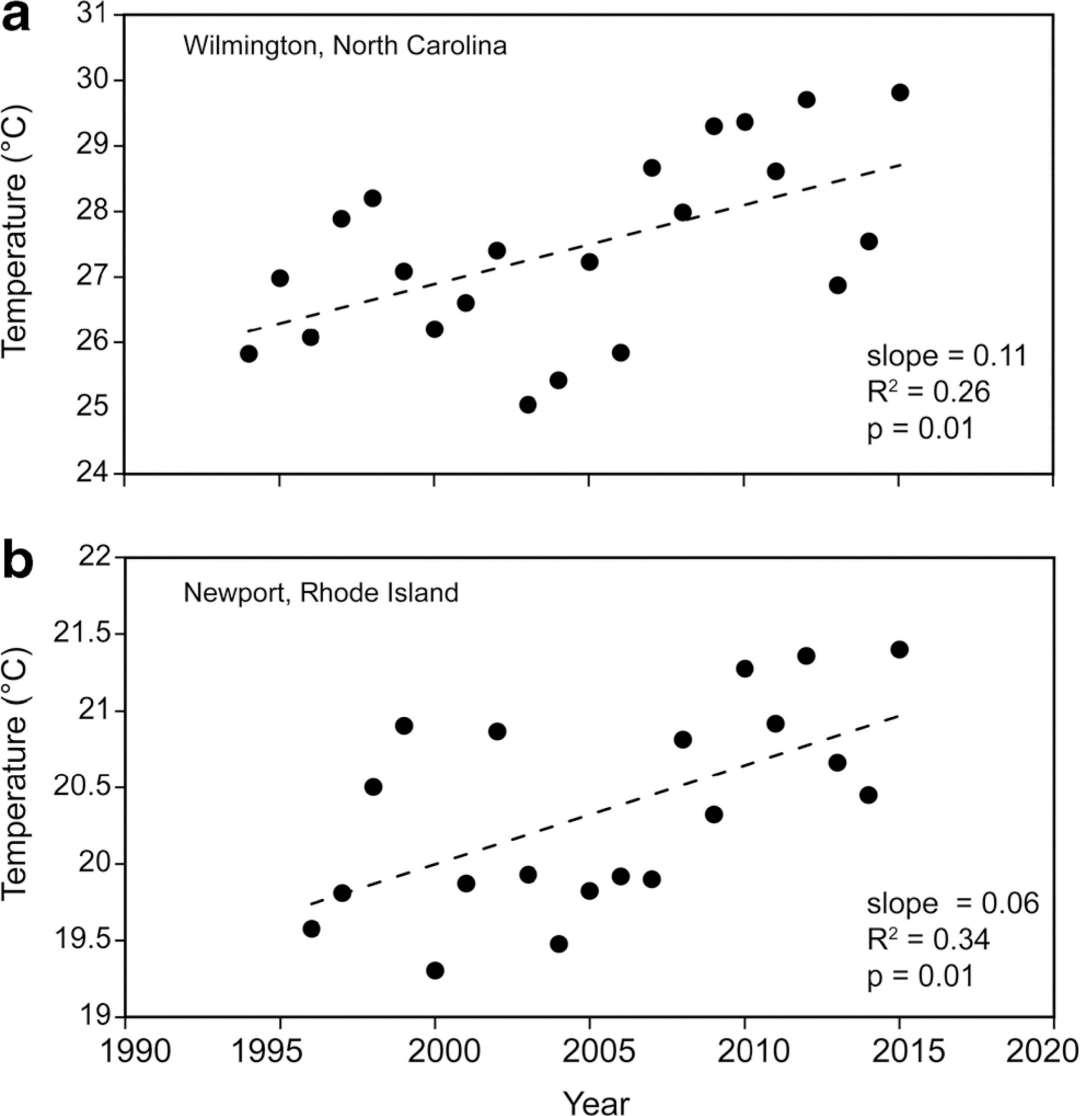
Time series for mean summer (July-September) water temperatures from National Oceanic and Atmospheric Administration tide gauges in the **a** Carolinian (Wilmington, NC) and **b** Virginian (Newport, RI) biogeographic provinces. Temperature sensors were about 1 m below mean lower low water Data begin at the point in the mid-1990s when NOAA switched from analog to digital temperature sensors

Both bottom and surface water temperatures in the combined dataset increased in all the 1° latitude bands from the earliest year of sampling to 2010, with more change in the lower latitudes (Table 2). The increase in mean surface water temperature was 1.7 °C (p < 0.001), and the increase in bottom water was 1.6 °C (p < 0.001). The Spearman correlations of both surface and bottom water temperatures, 1993/1994— 2010, showed a significant increase (p < 0.001); the warming trend can be seen in the mean values over time (Fig. 3). This is consistent with the trends in the region observed at the NOAA tide gauges (Fig. 4). Similar to our data, the CP station (Wilmington) in the NOAA data showed a greater increase (2.3 °C) than the 1.1 °C of the VP station (Newport; Fig. 4). Figures 3 and 4 show that the beginning and ending years of our combined dataset were not unusually cool or warm years.

### Changes in Species’ Centers of Abundance over Time

**Fig. 5:**
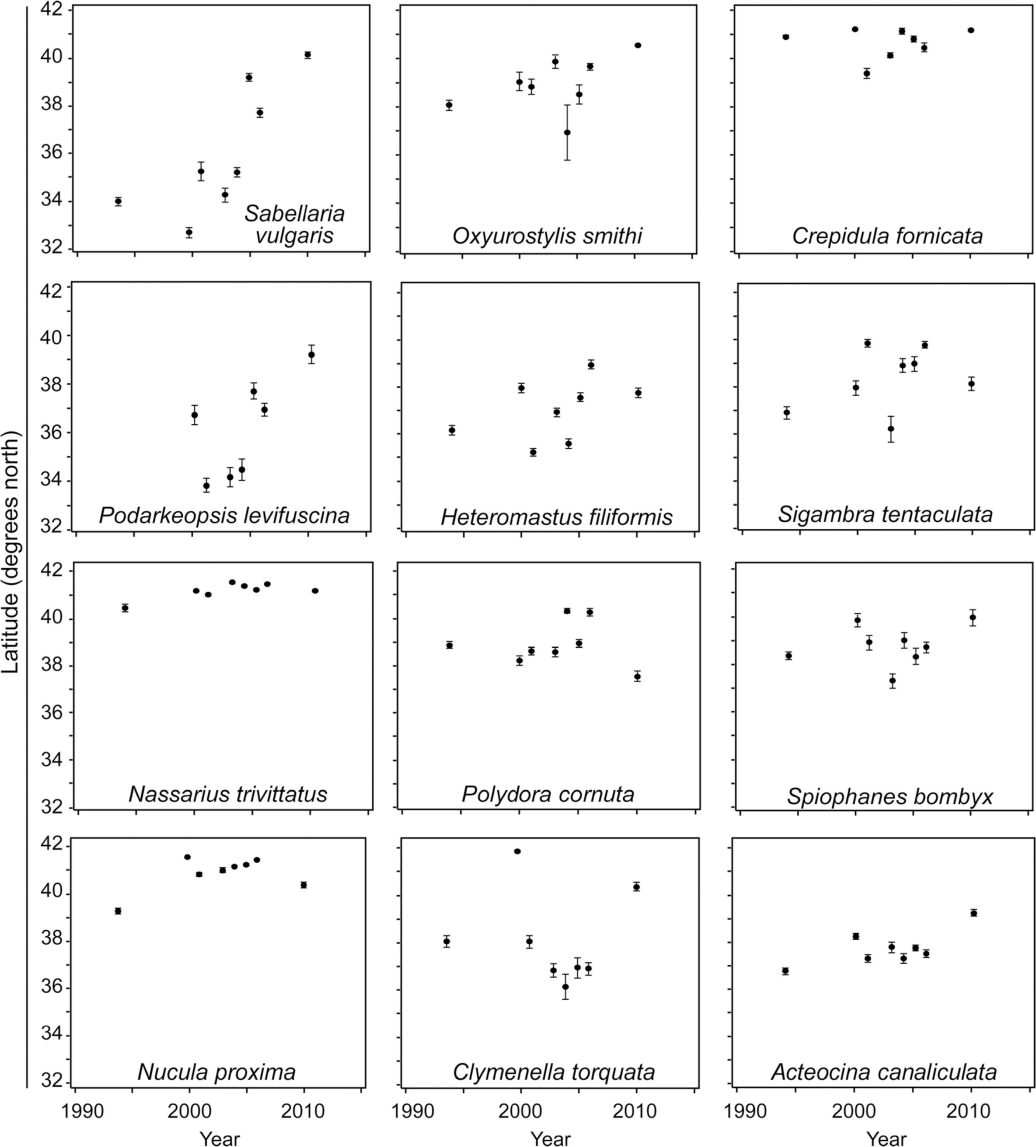
Centers of abundance (mean of log abundance-weighted latitudes of occurrence) by year of the 12 benthic species in the Carolinian and Virginian biogeographic provinces that showed the strongest Spearman correlations (>0.10) with year 1993/1994–2010

From the Spearman correlations of centers of abundance by year, of the 30 most prevalent species in the study area, 18 species (60%) showed significant (p < 0.01) shifts north in mean latitude during the period 1993/1994–2010 (Table 3). Seven (23%) showed significant movements south. Although the Spearman rank-order correlations of movement of species’ centers of abundance with year were relatively low because of year-to-year variance, 25 out of 30 were statistically significant (p < 0.01; Table 3), and a sign test (SAS, ver. 9.4) showed that 18 northward-movers out of 25 represented a significant shift (p = 0.02). Eleven of the 12 strongest Spearman correlations (0.10–0.51) were for species that shifted north; one was for a species that shifted south (Fig. 5; Table 3). Northward- moving species shifted their center of abundance 6–697 km (mean distance 181 km; rate 11 km year^−1^). These species came from three phyla and five different taxon groups (Table 3). Southward-moving species moved much shorter distances (mean 65 km, rate 4 km year ^1^). Some of the species in Fig. 5 showed a more northern center of abundance (e.g., *Nucula proxima),* while others were more ubiquitous (e.g., *Sabellaria vulgaris).*

### Changes in Species’ Minimum and Maximum Latitudes

From 1993/1994 to 2010, out of the 30 most prevalent species, 22 (73%) showed a northward shift in their southern extent (statistically significant from sign test at *p* = 0.01), with a mean shift north of 316 km (Table 4). At the northern extent, 20 species showed a northward shift, marginally significant at *p* = 0.07. These shifts resulted in an average range contraction of 25% for 20 species. There were also range expansions for 8 species; however, for the 30 species together, the mean range extent in the study area in 2010 was significantly smaller (*p* < 0.05) than in 1993/1994. Most of the range contractions were a result of a northward shift of the trailing edge that was not matched by a northward shift of the leading edge (Table 4). This point is reinforced by the significant 10th quantiles (Table 3), which indicate the northward trend of southern limits.

### Multidimensional Scaling of Abundances of All Species

**Fig. 6:**
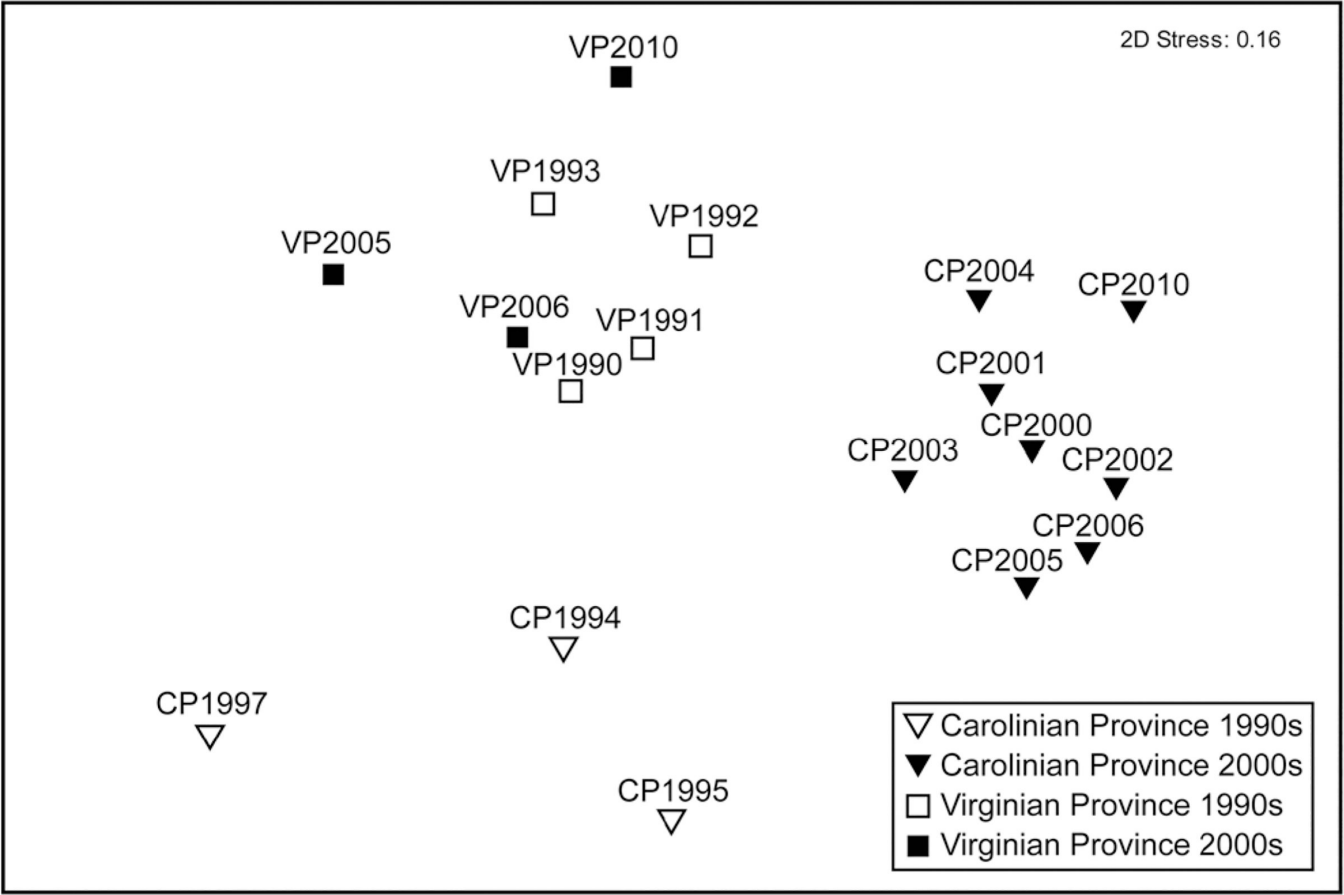
Multidimensional scaling of abundances of 1092 benthic species in the Carolinian (CP) and Virginian (VP) biogeographic provinces, 1990–2010. Axes are dimensionless. Points closer together are more similar in community composition, based on a Bray-Curtis similarity matrix

The plot (Fig. 6) shows that community composition changed over time, particularly in the CP. Stress is a measure of how well a two-dimensional plot represents a multivariate structure; a value of 0.16 suggests the plot is adequate but requires a look at the analysis of similarity (Clarke et al. 2014). As expected, the ANOSIM on mean species’ abundances showed that community compositions between the two biogeographic provinces in all years were significantly different (*p* < 0.001). Community composition in all the province-decade pairs was significantly different (*p* < 0.05), with the exception of the CP 1990s-VP 2000s pair (*p* = 0.10), where the R statistic—a measure of dissimilarity—was one of the lowest (Table 5). Community composition within the CP significantly changed from the 1990s to the 2000s, while the VP changed much less (R statistic = 0.43) (Fig. 6, Table 5).

### Species’ Shifts to a More Northerly Biogeographic Province

We identified five CP species that were not found in the VP in early sampling years but were found in the VP in 2010 and were not listed in WoRMS, OBIS, or the other species range databases as occurring this far north. Therefore, they potentially reflect range extensions. The species were the poly- chaetes *Magelona phyllisae, Pettiboneia duofurca, Scoletoma verrilli,* and *Thalassema hartmani* and the amphi- pod *Grandidierella bonnieroides.* These were relatively rare species (none in the 30 most common), with one to four records of occurrences and 6–15 individuals. WoRMS (2016) lists the distribution of these five species as Gulf of Mexico or Caribbean (*T. hartmani* also listed in western North Atlantic). There was no evidence that any VP species had moved south to the CP during the study period.

## Discussion

### Warming Waters

The mean temperature increase in the EPA data of 0.8–1.7 °C and the 1.1–2.3 °C at the NOAA tide gauges is greater than the global increase in the upper 75 m of the oceans, 1971–2010, of 0.11 °C decade^−1^ (IPCC 2014). There are several possible explanations for this. Globally, some of the fastest warming is occurring in the northwest Atlantic; the 2004–2012 rate of increase in the Gulf of Maine was 0.26 °C year^−1^ (Mills etal. 2013). Oczkowski etal. (2015) suggested that the effects of global warming can be amplified in shallow estuarine waters and found increases of 4–6 °C in intertidal estuarine waters in the northeast USA over the past four decades. Estuaries with large intertidal areas and shallow subtidal lighted bottoms may warm more than those without such areas. This may also be a partial explanation for the greater warming observed in the more southern latitudes in our data and at the NOAA tide gauges. Habitat differences such as large areas of shallow lagoons behind barrier beaches or extensive salt marshes may be a factor in regional differences. In other areas, studies (e.g., Pershing et al. 2015 in the Gulf of Maine; Sunday et al. 2015 in southeast Australia) have found regional warming differences attributable to climate change-induced shifts in ocean currents. Regarding future changes, Loarie et al. (2009) forecasted a higher rate of climate velocity in the southeast USA (up through Chesapeake Bay) than in the northeast.

### Species’ Range Shifts

Our results provide evidence that centers of abundance for 60% of the benthic species studied shifted north along the US Atlantic coast during the period 1990–2010, in concordance with increasing water temperatures. Further, the top 10 species with the highest correlations with year were all northward-movers. Species that shifted their centers of abundance north moved further than those that moved south. These changes occurred across five different taxonomic groups in three different phyla with a variety of life history strategies, feeding types, mobility, larval dispersal, and habitat preferences. The southern limits of 73% of the species studied shifted north; range contractions resulted when this was not accompanied by a northward shift of the northern limits. Community composition (drawing upon all 1092 species) in each province changed from the 1990s to the 2000s, more so in the southern latitudes. Finally, we found preliminary evidence that five CP species had surmounted their northern biogeographic boundary and extended their range into the VP, while no species had moved in the opposite direction. Even with small changes, persistent trends can lead to ecologically important consequences.

Our results are comparable to those found in similar subtidal, soft-bottom benthic studies. Hiddink et al. (2015) found that the distribution of 65 soft-bottom benthic invertebrates in the North Sea from 1986 to 2000, during which time mean bottom water temperature increased by 0.31 °C, showed significant poleward range shifts (distribution centroids, leading and trailing edges) in response to warming temperatures. There was evidence of species’ movements lagging behind concurrent shifts in water temperatures (Hiddink et al. 2015). Most species in that study shifted their centers of distribution at a rate of 4–7 km year ^1^, comparable with our mean rates of 4–11 km year ^1^. A review of studies of marine species responses to climate change over the period 1960 to 2009 reported poleward shifts of benthic invertebrates in the range of 0–150 km with some studies reporting shifts as great as 500–1000 km (Poloczanska et al. 2013).

The results of our analyses parallel studies of shifts of intertidal invertebrates and fishes along the US Atlantic coast. From 1968 to 2007, the center of biomass of 17 of 36 fish stocks on the northeast US continental shelf in the Acadian and Virginian provinces shifted north ~ 150–600 km (Nye et al. 2009). Similar to the pattern observed in our study, fish stocks in the southern end of the area exhibited more northward shift than those in the northern area. Nye et al. (2009) suggested that this may be a result of increases in temperature causing increased mortality at the southern extent of fish species’ ranges, particularly for early life stages. Since 1960, the southern extent of the intertidal barnacle, *Semibalanus balanoides*, in the VP has moved ~350 km north (Jones et al. 2012). Temperature at the southern end of the VP had increased enough to exceed the upper thermal tolerance of the barnacle, causing high rates of summer mortality; it is likely that temperature-dependent reproductive failure also contributed (Jones et al. 2012). The blue mussel, *Mytilus edulis,* showed a similar pattern (Jones et al. 2012). In the Mediterranean Sea, Rilov (2016) found evidence of multispecies collapses of abundant, non-harvested marine species (gastropods, other molluscs, sea urchins) at the warm edge of species’ distributions where populations are more vulnerable to stress.

The larger change in community composition in the CP than in the VP likely resulted from the greater warming in the CP, as evidenced by the EPA data and the NOAA tide gauge data. The lack of a significant difference in community composition of the VP in 2010 with that of the CP in 1994 is what would be expected if (1) species that existed in both provinces were becoming relatively less abundant in the CP and more abundant in the VP during the study period or (2) CP species that had not existed in the VP in the early years were appearing there in the later years. At the same time, the small and insignificant changes in northern limits in the study area may be a result of constraints imposed by the southern New England coastline and the sharp biogeographic boundary at Cape Cod. The study area used did not address the question of movements from the VP to the Acadian Biogeographic Province (where sampling did not begin until 2000).

The previously unrecorded occurrence in 2010 of five Gulf of Mexico and Caribbean species in the VP suggests that the waters had warmed enough for these species to surmount the northern biogeographic boundaries of the CP. Whether they are able to survive, grow, and reproduce there can be resolved with subsequent sampling. Such benthic species’ range extensions poleward beyond biogeographic boundaries have been noted elsewhere (e.g., Southward et al. 1995; Hawkins et al. 2008; Hinz et al. 2011; Johnson et al. 2011; Johnson 2014; Sunday et al. 2015).

In addition to temperature, benthic species’ distributions are also influenced by geography, ocean currents, ecological history, and interactions with other species (Briggs 1995; Hiscock et al. 2004; Johnson et al. 2011; Wernberg et al. 2012). Estuarine benthic species’ distributions in the area can be strongly influenced by salinity, sediment grain size, and percent organic matter, among other factors (e.g., Hale 2010). While these factors may influence where species become successfully established, rising temperature is the most plausible explanation for the northward shifts observed in three phyla throughout 15° of latitude in the present study (Briggs 1995; Hawkins et al. 2008; Wernberg et al. 2012; Pinsky et al. 2013; Hiddink et al. 2015; Sunday et al. 2015; Hare et al. 2016).

### Ecological Consequences of Species’ Range Shifts

The implications of the observed northward shifts of centers of abundance and the changes in range extents are that, over time, these processes will affect benthic community structure and biodiversity with impacts on ecosystem functioning (Schiel et al. 2004; Pinsky et al. 2013; Hiddink et al. 2015; Lord and Whitlatch 2015). Contractions of range extents could potentially reduce population sizes. At the ecosystem level, changes in area of thermal habitat can compress or stretch marine species assemblages, which could intensify or reduce species’ interactions (Kleisner et al. 2016). Small temperature increases in marine coastal areas can lead to major ecological consequences (Nixon et al. 2004; Oviatt 2004). The mean northward distance shifted in our study is about equal to the distance from Delaware Bay to the Hudson River estuary. Consequences can be important, especially when keystone or habitat-forming species are involved (Smale and Wernberg 2013). For example, the fiddler crab, *Uca pugnax,* which has recently moved into the Gulf of Maine, is an ecosystem engineer that can affect coastal wetland productivity, biogeochemistry, and sediment structure (Johnson 2014). As water temperatures continue to rise in the future, we speculate that more CP species and their centers of abundance will move to the VP, and the composition of species in the VP will to a greater extent resemble the 1994 CP.

These changes have implications for numerous ecosystem functions and services, including impacts to fisheries and the local economies that rely on them (Oviatt 2004; Nye et al. 2009; Johnson et al. 2011; Mills et al. 2013; Nelson et al. 2013; Pershing et al. 2015; Wahle et al. 2015; Hare et al. 2016). Changes to the mix of species in an estuary or coastal area can cause cascading ecological effects such as major changes in food webs and energy flow (Johnson et al. 2011) and have widespread socioeconomic consequences, e.g., diminished lobster fisheries in coastal southern New England (Wahle et al. 2015) and movement of southern fishes into the northern VP (Oviatt 2004). If benthic invertebrates do not move as rapidly as fish in response to climate warming, there is potential for a spatial mismatch between predator and prey. Fish species outpacing their familiar benthic prey poleward may have to switch to new sources, while lagging benthic species may experience a change of predators (e.g., Atlantic cod leaving the northern end of the VP and black sea bass moving in).

Our ability to document the ecological consequences of rising temperatures was limited by several factors. First, we limited our analysis to the 30 most common species. Less common species in the study area likely experienced range shifts as well, but it was not practical to test for movement of each of the 787 species because most of them were rare and we could not reliably rule out false absences. Second, species may have their regional distribution limits beyond our study area, and therefore, our reported distribution shifts are likely to be underestimates. Third, the northeast-trending coastline of the northern part of the VP, from New York Harbor to the southeast corner of Cape Cod, and the colder waters north of Cape Cod represent a significant barrier to range extension (Hale 2010). Whether and when the changes that we observed will necessitate a re-definition of current biogeographic boundaries remains to be seen. Cape Hatteras may be less of a barrier than Cape Cod for estuarine species because of the Albemarle-Pamlico Sound inside Cape Hatteras. However, Cape Cod has a north-south canal that has provided a shortcut to the Gulf of Maine for some species (Hale 2010).

Given a projected 1 −5 °C increase in ocean surface temperature on the Atlantic coast ofthe US by 2100 (IPCC 2014), and even greater increases likely in shallow estuarine areas (Oczkowski et al. 2015), more affected species and more range changes are to be expected in the future (Southward et al. 1995; Weinert et al. 2016). The continuing NCCA monitoring program will provide opportunities to repeat the analyses described herein with longer time series that will support more robust statistical analyses and conclusions. Expansion of the study area to include the Acadian Biogeographic Province (Gulf of Maine) will be of special interest because of the recent sharp increase in water temperatures there (Mills et al. 2013; Pershing et al. 2015). The relatively rapid climate change happening along the US Atlantic coast could lead to widespread species’ range shifts and is likely to have significant ecological and socioeconomic implications (Wernberg et al. 2012; Pershing et al. 2015; Hare et al. 2016). Monitoring programs that cover broad areas and track multiple species are crucial for predicting and documenting benthic community response to future warming, so resources can be managed appropriately (Hawkins et al. 2008; Hinz etal. 2011; Wernberg et al. 2012).

## Figures and Tables

**Table 1:** The 30 most prevalent (occurred at the most stations) species and their abundances in the Carolinian and Virginian biogeographic provinces, 1990–2010

Species (ITIS name)	WoRMS name	Total occur^a^	Total abund^b^
*Streblospio benedicti* (P)		357	27,714
*Mediomastus ambiseta* (P)		302	12,214
*Ampelisca abdita-vadorum* (A)		212	13,676
*Polydora cornuta* (P)		197	2575
*Tharyx acutus* (P)		194	7481
*Heteromastus filiformis* (P)		193	1408
*Acteocina canaliculata* (G)		190	2473
*Glycera americana* (P)		186	414
*Nucula proxima* (B)		181	10,978
*Tellina agilis* (B)	Ameritella agilis	177	3458
*Glycinde solitaria* (P)		159	923
*Paraprionospio pinnata* (P)		152	969
*Mulinia lateralis* (B)		151	2778
*Nephtys incisa* (P)		122	2108
*Scoloplos robustus* (P)	Leitoscoloplos robustus	117	1334
*Spiophanes bombyx* (P)		115	605
*Nassarius trivittatus* (G)	Tritia trivittata	110	673
*Pectinaria gouldii* (P)		110	561
*Edotia triloba* (I)		109	517
*Acmira catherinae* (P)	*Aricidea* (Acmira) *catherinae*	104	2799
*Nephtys picta* (P)		98	315
*Eusarsiella zostericola* (O)		95	951
*Crepidula fornicata* (G)		88	4741
*Podarkeopsis levifuscina* (P)		87	325
*Sigambra tentaculata* (P)		87	569
*Oxyurostylis smithi* (C)		83	241
*Sabellaria vulgaris* (P)		83	1874
*Neanthes succinea* (P)	Alitta succinea	82	627
*Clymenella torquata* (P)		81	584
*Gemma gemma* (B)		81	12,777

*ITIS* Integrated Taxonomic Information System, *WoRMS* World Register of Marine Species, *P* Polychaeta, *A* Amphipoda, *G* Gastropoda, *B* Bivalvia, I Isopoda, *O* Ostracoda, C Cumacea

a Total occurrences at 800 stations

b Total abundances, one 0.04-m^2^ grab per station; 0.5-mm sieve

**Table 2:** Mean surface and bottom temperatures in 1° latitude bands in the Carolinian (1994–2010) and Virginian (1990–2010) biogeographic provinces

Latitude band (°N)^a^	Surface temperature (°C)			Bottom temperature (°C)		
	First year	Last year	Change	First year	Last year	Change
27.0–28.0	−	−	−	−	−	−
28.0–29.0	29.5	31.5	2.0	28.8	31.3	2.5
29.0–30.0	−	−	−	−	−	−
30.0–31.0	28.1	30.2	2.1	27.8	29.3	1.5
31.0–32.0	28.2	31.4	3.2	27.8	31.2	3.4
32.0–33.0	27.7	30.1	2.4	27.8	30.7	2.9
33.0–34.0	27.6	29.2	1.6	27.1	29.4	2.3
34.0–35.0	28.3	28.9	0.6	28.0	29.0	1.0
35.0–36.0	26.5	28.4	1.9	26.4	27.5	1.1
36.0–37.0	26.8	28.9	2.1	27.1	27.8	0.7
37.0–38.0	26.7	27.4	0.7	26.5	26.9	0.4
38.0–39.0	25.8	26.4	0.6	25.7	25.9	0.2
39.0–40.0	24.6	25.2	0.6	24.7	25.1	0.4
40.0–41.0	23.1	25.9	2.8	22.6	24.8	2.2
41.0–42.0	21.6	22.7	1.1	20.0	21.9	1.9
Mean	26.5	28.2	1.7	26.2	27.8	1.6
Std error	0.6	0.7	0.2	0.7	0.8	0.3
*p*			<0.001			<0.001

a The two bands with missing values are in areas with fewer estuaries and long stretches of sandy beaches, resulting in <3 stations after standardizing stations by latitude

**Table 3:** The 25 species out of the 30 most prevalent (occurred at the most stations) that showed a significant change (*p* < 0.01) in mean centers of abundance in a Spearman correlation with year, Carolinian and Virginian biogeographic provinces, 1993/1994–2010

Species	Direction	Spearman correlation >0.10^a^	10th quantile significant at *p* <0.01^b^
*Acmira catherinae* (P)	North		
*Heteromastus filiformis* (P)	North	X	X
*Mediomastus ambiseta* (P)	North		
*Neanthes succinea* (P)	North		
*Podarkeopsis levifuscina* (P)	North	X	X
*Polydora cornuta* (P)	North	X	X
*Sabellaria vulgaris* (P)	North	X	X
*Sigambra tentaculata* (P)	North	X	
*Spiophanes bombyx* (P)	North	X	
*Tharyx acutus* (P)	North		
*Acteocina canaliculata* (G)	North	X	X
*Crepidula fornicata* (G	North	X	X
*Nassarius trivittatus* (G	North	X	
*Gemma gemma* (B)	North		
*Nucula proxima* (B)	North	X	X
*Tellina agilis* (B)	North		
*Edotia triloba* (I)	North		
*Oxyurostylis smithi* (C)	North	X	
*Clymenella torquata* (P)	South	X	
*Nephtys incisa* (P)	South		
*Pectinaria gouldii* (P)	South		
*Scoloplos robustus* (P)	South		
*Streblospio benedicti* (P)	South		
*Mulinia lateralis* (B)	South		
*Eusarsiella zostericola* (O)	South		

*P* Polychaeta, *G* Gastropoda, *B* Bivalvia, I Isopoda, C Cumacea, *O* Ostracoda

a From Spearman rank-order correlation between centers of abundance and year

b From quantile regression on centers of abundance and year; 90% of the observations were greater

**Table 4:** Number of species (out of the 30 most commonly occurring) in which the minimum or maximum extents in the Carolinian and Virginian biogeographic provinces shifted between the first (1993/1994) and last (2010) year of sampling

Parameter	Direction	No. of species (out of30)	Significance	Distance shifted	
				Range (km)	Mean (km)
Minimum latitude	N	22	*p* = 0.01	47–1051	316
	S	8	NS	−	−
Maximum latitude	N	20	*p* = 0.07	1–33	12
	S	10	NS	−	−

NS not significant

**Table 5:** Analysis of similarity (Bray-Curtis similarity matrix) of 1092 benthic species abundances in the Carolinian and Virginian biogeographical provinces, 1990–2010

Province-decade pair	*R* statistic	*p*
CP1990s-CP2000s	0.93	0.01
CP1990s-VP1990s	0.67	0.03
CP 1990s-VP2000s	0.59	0.10
CP2000s-VP1990s	0.87	0.01
CP2000s-VP2000s	0.97	0.01
VP 1990s-VP2000s	0.43	0.03
